# Current Epidemiological Status and Antibiotic Resistance Profile of *Serratia marcescens*

**DOI:** 10.3390/antibiotics13040323

**Published:** 2024-04-03

**Authors:** Ilaria Cosimato, Biagio Santella, Sandra Rufolo, Paola Sabatini, Massimiliano Galdiero, Mario Capunzo, Giovanni Boccia, Veronica Folliero, Gianluigi Franci

**Affiliations:** 1 U.O.C. of Virology and Microbiology, University Hospital “Luigi Vanvitelli”, 80138 Naples, Italy; ilaria.cosimato1@studenti.unicampania.it (I.C.);; 2 Department of Medicine, Surgery and Dentistry “Scuola Medica Salernitana”, University of Salerno, 84081 Baronissi, Italy; bi.santella@gmail.com (B.S.); s.rufolo2@studenti.unisa.it (S.R.); mcapunzo@unisa.it (M.C.); gboccia@unisa.it (G.B.); vfolliero@unisa.it (V.F.); 3 Unit of Virology and Microbiology, Nocera Inferiore, Umberto I Hospital, 84018 Salerno, Italy; p.sabatini@aslsalerno.it; 4 Dai Dipartimento Di Igiene Sanitaria e Medicina Valutativa, U.O.C. Patologia Clinica e Microbiologia, A.O.U. San Giovanni di Dio e Ruggi D’Aragona, Largo Città di Ippocrate, 84131 Salerno, Italy

**Keywords:** *Serratia marcescens*, antibiotics, Gram-negative bacteria, susceptibility patterns, multidrug resistance

## Abstract

The spread of antibiotic resistance represents a serious worldwide public health issue, underscoring the importance of epidemiology research in determining antimicrobial strategies. The purpose of this research was to investigate antibiotic resistance in *Serratia marcescens* isolates from clinical samples over seven years at the University Hospital “San Giovanni di Dio e Ruggi d’Aragona” in Salerno, Italy. *S. marcescens* is an important opportunistic pathogen associated with a wide spectrum of clinical diseases, including pneumonia, keratitis, meningitis, and urinary tract and wound infections. Outbreaks of nosocomial infections by *S. marcescens* strains have been documented in high-risk settings, mainly affecting immunocompromised patients and newborns. The primary objective of this study is to assess the rates of antibiotic resistance over the years to deal with a future emergency which includes the failure of various therapies due to antibiotic resistance. During the investigation, a total of 396 species of *S. marcescens* were isolated from various clinical samples, mainly from broncho-aspirates and sputum (31.6%) and blood cultures (21.5%). Antibiotics that showed the greatest susceptibility included ceftazidime/avibactam, amikacin, trimethoprim/sulfamethoxazole, and selected members of the cephalosporin class. However, a disconcerting trend of increasing rates of carbapenem resistance was outlined over the observation period. The absence of effective countermeasures, combined with growing antibiotic resistance that negates the effectiveness of multiple antibiotics, highlights the potential for *S. marcescens* infections to trigger serious clinical complications and increased mortality rates. The surveillance of *Serratia marcescens* infections constitutes a pivotal element in refining empiric therapy to mitigate the dissemination of antimicrobial resistance.

## 1. Introduction

*Serratia marcescens* (*S. marcescens*) is a Gram-negative bacillus belonging to the *Enterobacteriaceae* family, commonly found in environments such as water, plants, and soil. Recent studies have emphasized the threat posed by this species that has emerged as a relevant opportunistic pathogen, prominently reported in nosocomial outbreaks in neonatal intensive care units (NICUs), intensive care units (ICUs), and various hospital settings [1]. This strain demonstrates remarkable pathogenic versatility, capable of eliciting a broad spectrum of clinical pathologies, which include wounds, urinary tract infections, central nervous system infections, bloodstream infections, pneumonia, and keratitis. The incidence of infections attributed to *S. marcescens* is linked to the expression of numerous virulence factors and the emergence of antibiotic resistance. Several factors contribute to the opportunistic nature of this bacterium, including compromised clinical conditions of patients, prolonged hospitalization, exposure to medical interventions, and the increased frequency and intensity of direct contact with healthcare personnel. This highlights the increased susceptibility of patients to *S. marcescens* infections in healthcare settings and highlights the need for careful monitoring and preventative measures to curb the spread of this pathogen.

In Europe, data from the European Centre for Disease Prevention and Control (ECDC) place *Serratia* spp. in the sixth position among the main microorganisms isolated in patients with pneumonia admitted to the UTI in 2016 [2]. Similarly, it ranks in the tenth and ninth position for bloodstream and urinary infections, respectively (ECDC, 2018) [3]. In Italy, similar data were reported by the Project Prospective Surveillance of Nosocomial Infections in the SPIN-UTI study, in which *S. marcescens* ranks seventh among the microorganisms isolated in pneumonia and ninth in central venous catheter-associated bloodstream infections [4]. More evidence increasingly reports *S. marcescens*-related epidemic episodes, further complicated by the spread of antibiotic-resistant strains [5]. The group “ESCPM”, which contains *S. marcescens*, *Enterobacter*, *Citrobacter freundii*, *Providencia*, and *Morganella morganii*, exhibits elevated levels of AmpC expression. *S. marcescens* carries a chromosomally encoded ampC gene, which contributes to an extensive resistance profile that includes various β-lactam antibiotics [6,7]. According to the existing literature, *S. marcescens* exhibits resistance to a broad spectrum of antibiotics, encompassing penicillin, cephalosporin, tetracycline, macrolide, nitrofurantoin, and colistin [8].

Furthermore, the production of deoxyribonuclease (DNase), lipase, and gelatinase makes this species capable of resisting different classes of antibiotics. In addition, it produces ShIA, a hemolysin that forms pores and has the potential to induce cellular damage and release inflammatory mediators [9]. Previously, aminoglycosides, fluoroquinolones, and third generation cephalosporins were the cornerstones of *S. marcescens* infection treatment. The early 1990s witnessed the documentation of the significant efficacy of fluoroquinolones against *S. marcescens*, as evidenced by the minimum inhibitory concentrations (MICs) of 0.5 µg/mL for ciprofloxacin and levofloxacin. In recent years, however, the incidence of *S. marcescens* resistant to fluoroquinolones has increased due to the increased use of these drugs in the treatment of hospitalized patients [10].

Furthermore, carbapenems constitute an effective alternative treatment because they remain active against bacteria that express high levels of AmpC and ESBLs. However, the emergence of carbapenemase-mediated resistance in *S. marcescens* suggests a reduction in the use of these agents. Considering the continuing evidence of hospital infections associated with *S. marcescens*, it is evident that infection control opportunities depend not only on the prudent use of antimicrobials but also on the implementation of effective infection control policies [11].

This scenario represents a challenge to antibiotic resistance and a starting point for evaluating new clinical and pharmaceutical strategies to fight the spread of increasingly dangerous bacterial strains. An antimicrobial resistance surveillance study conducted in medical centers and regional hospitals in Taiwan from 2002 to 2010 highlights a high susceptibility to ceftazidime and a high resistance to ciprofloxacin and levofloxacin, emphasizing that the utility of continuous monitoring, especially for fluoroquinolones, is necessary [12].

The objectives of our study were to analyze the antibiotic resistance profiles associated with *S. marcescens* species isolated from clinical specimens in a hospital in southern Italy between 2015 and 2022, reporting the change in resistance rates to different classes of antibiotics tested. The results will be useful to support targeted and efficient antibiotic therapy and improve empirical therapy.

## 2. Results

**Figure 1:**
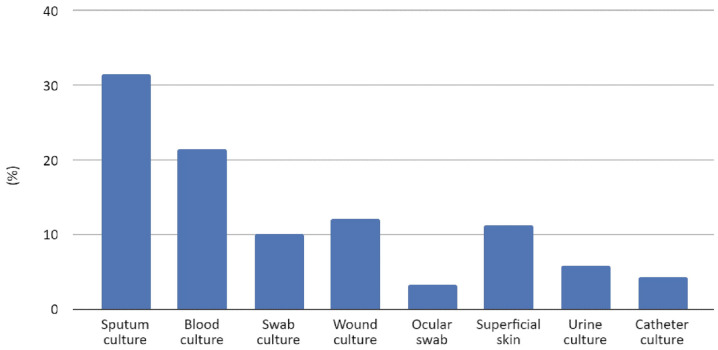
Clinical sample types and rates of *S. marcescens* isolates.

From January 2015 to December 2022, the strains of *S. marcescens* isolates from clinical samples at the University Hospital “San Giovanni di Dio e Ruggi d’Aragona” in Salerno were analyzed. During the investigation, a total of 396 species of *S. marcescens* were isolated from various clinical specimens. Specifically, these species were isolated from clinical samples: 125 from broncho-aspirates and expectorates (31.6%), 85 from blood cultures (21.5%), 48 from wound cultures (12.1%), 45 from skin (11.4%), 40 from sternal swabs (10.1%), 23 from urine cultures (5.8%), 17 from catheters (4.3%), and 13 from ocular swabs (3.3%), as reported in Figure 1. Throughout the study period, the isolation rate remained relatively consistent. *Serratia* species were predominantly isolated from blood samples and lower respiratory tract specimens. Notably, a higher number of isolates were observed in the last two years of the study (2021–2022), nearly doubling compared to preceding years (Table S1).

Over the course of the study spanning several years, the resistance patterns of antibiotics within the penicillin class revealed notable trends (Table 1). Initially, amoxicillin/clavulanic acid exhibited a resistance rate of 89.1% in the first year, with subsequent years showing an upward trajectory in resistance. Ceftazidime/avibactam emerged as a standout with the lowest resistance rates, followed by amikacin and trimethoprim/sulfamethoxazole, as detailed in Table S2. Within the cephalosporin class, resistance rates were observed at 20.7% for ceftazidime, 25.3% for cefotaxime, and a reduced rate of 8% for cefepime in 2022. The carbapenem class, in its inaugural year, showcased a 4.2% resistance rate for meropenem. However, variations unfolded over subsequent years, reaching peaks of 7.5% for meropenem in 2020. In the realm of fluoroquinolone-class antibiotics, ciprofloxacin exhibited fluctuating resistance rates over the years, ranging from 2.1% in 2015 to 20.4% in 2019 and peaking at 39.7% in 2021. Lastly, trimethoprim/sulfamethoxazole maintained a stable resistance rate around 5.7%.

## 3. Discussion

In our study, *S. marcescens* was isolated mainly from lower respiratory tract samples (31.6%), followed by blood cultures (21.5%); these data underline its ability to cause pneumonia and sepsis. Moreover, it was identified from urine samples (5.8%) and catheters (4.3%) less frequently, indicating a reduced pathogenicity in the urinary system.

Consistent with our finding, the study published by Bo Liu et al. in China on 937 strains collected from 2014 to 2020 reported a higher prevalence of isolates from respiratory samples (79.3%). This proportion markedly exceeded the study by Bo-Huang et al., which involved 157 strains recovered from respiratory samples (39.0%) [13,14].

Similarly, a 2018 study in Turkey, analyzing 158 clinical specimens, noted a common isolation of *S*. *marcescens* in blood culture (35.4%), sputum culture (24.6%), and wound cultures (15.8%) [15]. Furthermore, a 2015 Polish investigation on 81 strains of *S. marcescens* by Młynarczyk G. et al. revealed a higher prevalence of isolates from urine (70%) than previous studies, followed by respiratory tract samples (4%) and blood cultures (8%) [16].

Moreover, in the last two years of our study (2021–2022), the number of *S. marcescens* isolates increased by approximately double compared to previous years (2015–2020), with an increasingly higher prevalence of respiratory and blood culture samples.

According to our study, Şimşek M. et al. found that *Serratia* spp. strain isolates were sensitive to amikacin 95.7%, ciprofloxacin 91.5%, and gentamicin 82.2%; in fact, in our study, amikacin has a low resistance rate of 4.4%, ciprofloxacin of 20.7%, and gentamicin of 6.8% [15].

Other studies highlight the resistance of *S. marcescens* strains to beta-lactam and especially cephalosporin antibiotics, necessitating treatment decision on antibiotic susceptibility tests.

In the Turkish study, clinical isolates of *Serratia* spp. exhibited a high resistance to ceftriaxone, ceftazidime, and piperacillin/tazobactam [15]. However, cefotaxime and gentamicin were the most effective antibiotics suitable for treatment. In our study, we found that the antibiotic ceftazidime, in agreement with the study, has a resistance rate of 20.2%. We also noticed a higher resistance rate for ceftolozane/tazobactam of 26.7%. Notably, the highest resistance rate concerns the amoxicillin/clavulanic acid combination, probably due to the widespread use of this antibiotic in nosocomial settings. In accordance with our data, Slain et al. demonstrated markedly elevated levels of resistance in strains of *S. marcescens* implicated in cases of bacteremia and endocarditis [17].

In a study from a tertiary hospital in China, spanning seven years of *S. marcescens* surveillance, resistance rates to meropenem declined significantly from 16.22% to 1.71% in 2019 and 2020 [13]. The researchers reached the conclusion that the strict prevention and control measures due to the COVID-19 pandemic may have played a role in the decreasing rates. Similarly, our study observed a low rate of resistance in 2019 for meropenem (6.1%), increasing slightly in 2020 to 7.5%.

The researchers in the Chinese study attributed the decrease to the strict prevention and control measures during the COVID-19 pandemic, a trend echoed in our study.

In Mexico, several nosocomial outbreaks due to *S. marcescens* have been reported. A study on 193 clinical isolates collected in 2016–2017 from eight medical centers in two regions in Mexico found amikacin susceptibility at 75.6% [18]. Worldwide, including Asia and Africa, amikacin remains highly active against *S. marcescens*, with susceptibility rates between 93 and 100%. Our study aligns with these findings, with amikacin demonstrating a low resistance rate of 4%. Furthermore, compared to the Mexican study reporting a ciprofloxacin susceptibility of 78.7%, our data show a lower resistance rate of 20.7%.

In another study, spanning from 2002 to 2010 across medical centers in Taiwan, *S. marcescens* isolates displayed varying susceptibility rates to different antibiotics as part of Taiwan’s antimicrobial resistance surveillance program [14]. A total of 403 *S. marcescens* isolates were collected, mostly from respiratory samples (157, 39.0%), followed by urinary tract samples (90, 22.3%). Our study aligns with low resistance rates for amikacin and cefepime. However, ciprofloxacin, with an increase in resistance over the years in our study (2.1% to 20.7%), exhibited a different trend compared to the Taiwanese study, which reported an increased sensitivity rate after 2004 (53.8% vs. 70.4%). The lower sensitivity for trimethoprim/sulfamethoxazole (TMP/SMX) and gentamicin in our study may be explained by differences in the types of samples collected. In fact, in this study conducted in Taiwan, many strains were isolated from urinary tract samples [14]. It is hypothesized that these infections were treated mainly with these antibiotics that acquired increased resistance over the years.

In another study conducted at the orthopedic clinic of the University of Sarajevo clinical center, 96 strains from 79 patients were isolated from January to December 2010 [19]. All patients had episodes of clinically significant infection and required therapy with antibiotics. There were 17 episodes of septicemia, 1 case of meningitis, and 78 wound infections. Strains were isolated from wound swabs (81%), blood cultures (18%), and cerebrospinal fluid (1%). The isolates showed resistance to amoxicillin/clavulanic acid and to cefotaxime, ceftazidime, cefepime, trimethoprim/sulfamethoxazole, and gentamicin. Isolates remained susceptible to meropenem, amikacin, ciprofloxacin, and piperacillin/tazobactam. This agrees with our study where we have a high resistance rate for amoxicillin/clavulanic acid and resistance rates of 24.5% and 20.2% for cefotaxime and ceftazidime, respectively. Cefepime, TMP/SMX, and gentamicin had the lowest resistance rates in our study: 9%, 5.3%, and 6.8%, respectively. Meropenem had low resistance rates in agreement with this study, while ciprofloxacin and piperacillin/tazobactam had resistance rates of 20.7% and 14.2%, respectively.

An intrinsic limitation of our investigation lies in the limited examination of sensitivity to the myriad of antibiotics used in clinical settings. This limitation arises from the use of automated systems for analysis, which are standard practice in our institution’s clinical microbiology laboratory. The conventional reference method used in our study allowed for only a subset of antibiotics to be tested. Furthermore, some antibiotics tested, notably colistin and fosfomycin, were omitted from the analysis, requiring further examination and validation through an additional method (manual microdilution in standard broth). This additional testing protocol was conducted solely upon specific request. However, this retrospective study presents the results provided to doctors with the aim of establishing an optimal therapeutic regimen to effectively address infections induced by this pathogenic species. Our study utilized the Vitek 2 system, which is integrated with the updated breakpoints provided by EUCAST. Neglecting to update these breakpoints could lead to changes in the way bacteria are classified, thus altering the levels of resistance to some antibiotics (fluoroquinolones, piperacillin/tazobactam, and various cephalosporins) reported in epidemiological studies. Here are how changes to EUCAST breakpoints can affect epidemiological resistance data: (i) The reclassification of isolates: updated breakpoints can reclassify isolates previously classified as susceptible, intermediate, or resistant or vice versa. This reclassification could impact the overall percentage of resistant isolates reported in epidemiological studies; (ii) impact on treatment guidelines: changes to breakpoints may influence antibiotic treatment guidelines. If breakpoints are lowered (i.e., become more stringent), this may indicate increased resistance and the need for adjustments in antibiotic prescribing practices; (iii) a comparison between studies: updated breakpoints can make it difficult to compare resistance rates between different studies, particularly if the studies use different versions of the breakpoints; (iv) clinical implications: changes in resistance data resulting from updated breakpoints may have clinical implications, influencing the selection of antibiotics for empiric therapy and patient management strategies; and (v) research and development: updated breakpoints may also stimulate research and development efforts for new antibiotics or alternative therapeutic strategies in response to emerging resistance patterns identified through surveillance data.

## 4. Materials and Methods

### 4.1. Sample Collection

The antibiotic susceptibility results of *S. marcescens* strains isolated from different clinical samples at A.O.U. San Giovanni di Dio e Ruggi D’Aragona in Salerno (Italy) between 2015 and 2022 were evaluated in this study. The samples included blood cultures, sputum cultures, wound cultures, urine cultures, tracheal aspirates, tissue cultures, and others (superficial skin samples, aspirate cultures, catheter cultures, and pleural cultures). The samples were analyzed in the bacteriology laboratory according to the guidelines for clinical bacteriology, which are described briefly below. The data were processed with the exclusion of redundant results, i.e., isolates of the same pathogen obtained from the same biological material in the 30 days following the first isolation for each patient were eliminated.

### 4.2. Identification and Antimicrobial Susceptibility Testing

All samples were collected in sterile containers and subjected to analysis within the microbiology laboratory, for strain isolation, identification, and evaluation of susceptibility tests, scrupulously adhering to the guidelines outlined by the European Committee on Antimicrobial Susceptibility Testing (EUCAST) [20,21,22].

Antimicrobial profiling and identification assays were conducted on *Serratia* spp. species isolated on blood agar. Identifications and antibiotic susceptibility were performed with VITEK 2 (bioMérieux, Inc., Hazelwood, MO, USA), a fully automated identification and antibiogram system, using an identification card (ID-GN) and susceptibility cards (AST-379 and AST-397, Gram-negative non-fermenters), according to the manufacturer’s instructions.

Pure cultures of bacteria grown on plates were used to create a bacterial suspension. Bacterial colonies were suspended in 3 mL of a 0.45% sodium chloride solution. A Densichek (bioMérieux, Marcy l’Etoile, France) was employed to adjust the bacterial suspension to a McFarland standard of 0.5. The antimicrobial susceptibility results were interpreted as “susceptible”, “resistant”, or “intermediate” according to EUCAST [23].

The Quality Control of Gram-negative (GN) cards was performed using two strains: *Enterobacter* ATCC 700.323 and *Klebsiella oxytoca* ATCC 700.324 for GN.

The following antibiotics (bioMerieux, Marcy l’Etoile, France) were included in the present study: amikacin (AK), amoxicillin/clavulanic acid (XL), cefepime (PM), cefotaxime (CT), ceftazidime (TZ), ciprofloxacin (CI), gentamicin (GM), meropenem (MP), piperacillin/tazobactam (P/T), trimethoprim/sulfamethoxazole (T/S), ceftazidime/avibactam (CZA), and ceftolozane/tazobactam (C/T).

### 4.3. Statistical Analysis

An Excel database was constructed of every sample that was evaluated during the years (Excel 2021, Microsoft Office Professional Plus 2021, Microsoft Corporation, Redmond, Washington, USA.). The resistance trend to antibiotics was analyzed using XLSTAT (XLSTAT statistical software for Microsoft Excel, v2023.3.1.1416 (32 bit), Lumivero). The Cochran–Armitage test for trend was applied to evaluate the trend in resistance for each antibiotic. An alpha equal to 5% was considered for the test; therefore, those associations with a *p*-value < 0.05 were considered statistically significant.

## 5. Conclusions

Surveillance studies play a crucial role in guiding antimicrobial use, particularly in shaping empirical therapy based on the local or regional resistance patterns of microorganisms. They also contribute to the implementation of infection control measures and supporting antibiotic stewardship efforts. Hand hygiene remains a cornerstone in infection control, with particular emphasis on reiterating its importance to all healthcare personnel when dealing with *S. marcescens*. Consideration may be given to grouping patients into specific rooms or units to minimize contact between staff and non-infected patients, with careful attention to isolation measures. In different studies, the reason for the presence of more resistant Serratia strains is usually the isolation of bacteria from the same clone that causes hospital infections during outbreaks. If we consider immunocompromised patients, elderly people, and newborns in intensive care or neonatal intensive care infected with this bacterium, it becomes clear that effective and correct antibiotic combinations are essential for treatment. Antibiotic susceptibility tests must be applied for the efficient treatment of patients with infections associated with *S. marcescens*. Treatment with an antibiotic combination considering the results of an antibiotic susceptibility test is of great importance for the successful treatment and eradication of an outbreak, depending on the infections, and to avoid the development of nosocomial infections that can lead to an increase in the cases of sepsis and the mortality rate. Health surveillance, the continuous monitoring of the antibiotic resistance rate, professional training courses, hygiene, and prevention are the only weapons available that can allow us to face these health emergencies that may not be alarming here and now but could be in the immediate future. 

## Figures and Tables

**Table 1:** The antibiotic resistance rates of S. marcescens strains over the years; R% (n. of isolates tested). NA (not assayed).

Antibiotics	2015	2016	2017	2018	2019	2020	2021	2022	Trend Analysis
Amikacin	4.2 (48)	4.0 (25)	0 (31)	2.9 (34)	6.4 (47)	5.4 (37)	3.8 (78)	5.7 (87)	*p* = 0.472
Amoxicillin/Clav. acid	89.1 (46)	100 (24)	100 (23)	100 (27)	100 (60)	100 (33)	100 (76)	100 (87)	*p* < 0.001
Cefepime	4.2 (48)	0 (26)	18.8 (32)	9.1 (33)	23.1 (13)	13.5 (37)	7.7 (78)	8.0 (87)	*p* = 0.601
Cefotaxime	12.5 (48)	3.8 (26)	25.0 (32)	5.6 (36)	22.9 (48)	25.0 (40)	51.5 (66)	25.3 (87)	*p* < 0.001
Ceftazidime	10.4 (48)	3.8 (26)	21.9 (32)	5.6 (36)	18.4 (49)	17.5 (40)	39.7 (78)	20.7 (87)	*p* < 0.001
Ciprofloxacin	2.1 (48)	3.8 (26)	25.0 (32)	8.3 (36)	20.4 (49)	25.0 (40)	39.7 (78)	20.7 (87)	*p* < 0.001
Gentamicin	6.3 (48)	0 (26)	12.5 (32)	8.3 (36)	4.1 (49)	10.0 (40)	3.8 (78)	9.2 (87)	*p* = 0.674
Meropenem	4.2 (48)	0 (26)	6.3 (32)	0 (36)	6.1 (49)	7.5 (40)	2.9 (69)	8.0 (87)	*p* = 0.612
Piperacillin/Tazobactam	6.3 (48)	0 (26)	14.3 (14)	0 (32)	14.3 (49)	10.0 (40)	25.6 (78)	19.5 (87)	*p* < 0.001
Trimethoprim/Sulfam.	6.3 (48)	3.8 (26)	15.6 (32)	2.8 (36)	0 (49)	7.5 (40)	3.8 (78)	5.7 (87)	*p* = 0.537
Ceftazidime/Avibactam	NA	NA	NA	NA	NA	6.9 (29)	3.0 (67)	5.0 (80)	*p* = 0.875
Ceftolozane/Tazobactam	NA	NA	NA	NA	NA	17.2 (29)	37.3 (67)	21.3 (80)	*p* = 0.706

## Data Availability

Data are contained within the article and Supplementary Materials.

## Supplementary Materials

The following supporting information can be downloaded at https://www.mdpi.com/article/10.3390/antibiotics13040323/s1, Table S1: Clinical sample types and rates of *S. marcescens* isolates in years; Table S2: The antibiotic resistance rates of *S. marcescens* strains.
