# Supplementary material for: Current Epidemiological Status and Antibiotic Resistance Profile of Serratia marcescens
Source: Antibiotics (Basel). 2024 Apr 3;13(4):323. doi: 10.3390/antibiotics13040323 (PMC11047617; doi:10.3390/antibiotics13040323)
Supplement: Supplementary file 1 [file antibiotics-13-00323-s001.zip › antibiotics-2876144-supplementary.pdf]

Table S1: clinical sample types and rates of *S. marcescens* isolates in years.

Clinical sample types	2015	2016	2017	2018	2019	2020	2021	2022
Sputum culture	23(47.9)	11(42.3)	6(18.8)	14(38.9)	16(32.7)	10(25.0)	15(19.2)	30(34.5)
Blood culture	5(10.4)	5(19.2)	7(21.9)	8(22.2)	6(12.2)	10(25.0)	21(26.9)	23(26.4)
Swab culture	3(6.3)	1(3.8)	3(9.4)	4(11.1)	4(8.2)	2(5.0)	12(15.4)	11(12.6)
Wound culture	5(10.4)	3(11.5)	8(25.0)	2(5.6)	12(24.5)	3(7.5)	10(12.8)	5(5.7)
Ocular swab	5(10.4)	0(0)	2(6.3)	1(2.8)	0(0)	3(7.5)	1(1.3)	1(1.1)
Superficial skin	4(8.3)	2(7.7)	0(0)	5(13.9)	7(14.3)	5(12.5)	13(16.7)	9(10.3)
Urine culture	1(2.1)	1(3.8)	6(18.8)	1(2.8)	3(6.1)	6(15.0)	5(6.4)	0(0)
Catheter culture	2(4.2)	3(11.5)	0(0)	1(2.8)	1(2.0)	1(2.5)	1(1.3)	8(9.2)
Total	48	26	32	36	49	40	78	87

Table S2: the antibiotic resistance rates of *S. marcescens* strains.

**2015- 2022**			
**Antibiotics**	**n. isolates resistance**	**n. isolates assay**	**% R**
Amikacin	17	387	4.4
Amoxicillin/Clav. acid	371	376	97.8
Cefepime	32	354	9.0
Cefotaxime	94	383	24.5
Ceftazidime	80	396	20.2
Ciprofloxacin	82	396	20.7
Gentamicin	27	396	6.8
Meropenem	19	396	4.7
Piperacillin/Tazobactam	53	374	14.2
Trimethoprim/Sulfam.	21	396	5.3
Ceftazidime\Avibactam	8	176	4.5
Ceftolozane/Tazobactam	47	176	26.7
